# Allostatic Load and Effort-Reward Imbalance: Associations over the Working-Career

**DOI:** 10.3390/ijerph15020191

**Published:** 2018-01-24

**Authors:** José Ignacio Cuitún Coronado, Tarani Chandola, Andrew Steptoe

**Affiliations:** 1 Cathie Marsh Institute and Social Statistics, University of Manchester, Humanities Bridgeford Street, Manchester M13 9PL, UK; jose.cuituncoronado@manchester.ac.uk; 2 Research Department of Behavioural Science and Health, University College London, London WC1E 6BT, UK; a.steptoe@ucl.ac.uk

**Keywords:** effort-reward imbalance, allostatic load, work stress, lifecourse

## Abstract

Although associations between work stressors and stress-related biomarkers have been reported in cross-sectional studies, the use of single time measurements of work stressors could be one of the reasons for inconsistent associations. This study examines whether repeated reports of work stress towards the end of the working career predicts allostatic load, a measure of chronic stress related physiological processes. Data from waves 2 to 6 of the English Longitudinal Study of Ageing (ELSA) were analysed, with a main analytical sample of 2663 older adults (aged 50+) who had at least one measurement of effort-reward imbalance between waves 2–6 and a measurement of allostatic load at wave 6. Cumulative work stress over waves 2–6 were measured by the effort-reward imbalance model. ELSA respondents who had reported two or more occasions of imbalance had a higher (0.3) estimate of the allostatic load index than those who did not report any imbalance, controlling for a range of health and socio-demographic factors, as well as allostatic load at baseline. More recent reports of imbalance were significantly associated with a higher allostatic load index, whereas reports of imbalance from earlier waves of ELSA were not. The accumulation of work related stressors could have adverse effects on chronic stress biological processes.

## 1. Introduction

Work-related stress is one of the key factors resulting in negative employee outcomes such as mental ill-health (e.g., depressive disorders, anxiety, and other stress related conditions) [1,2], disability, and early labour market exit [3]. Biological stress processes are suggested to underpin the associations between work related stressors and health [4,5]. However, much of the research on the biological consequences of work related stress has been cross-sectional [5], with almost no studies that have examined the cumulative effect of work related stressors over a working lifetime on biological stress processes. If greater exposure to work-related stressors over a working lifetime is associated with more adverse levels of biological stress responses, then this is stronger evidence that work-related stress negatively affects physiological health compared to cross-sectional associations [6].

In a meta-analysis of the association between job strain and coronary heart disease, Kivimäki, Nyberg, Batty, Fransson, et al. [7], found a small effect, with a hazard ratio of 1.23 (1.10–1.37) for incident coronary heart disease among workers with and without job strain. However, most of the component studies of the meta-analyses were based on a single measurement episode of work stress. Other studies using cumulative reports of work related stress tend to show stronger effects on health [8]. Research on life-course processes tends to emphasise the importance of accumulation and duration of disadvantages throughout the life course, as well as the timing of exposures within individual life courses. However, much of the published research on work stress and health do not take into account the accumulation, ordering, and embeddedness of work stress exposures within larger employment trajectories [9].

There are two main models used in the work stress studies, the job demand control, which postulates that lower control and higher work demands can trigger job strain [10], and the effort-reward imbalance (ERI) model that emphasizes social reciprocity [11]. In the ERI model, when there is a sustained unfair trade-off between effort and reward, negative emotions can be elicited, which can lead to adverse long-term health consequences [12]. Psychosocial stress at work is experienced as a result of challenges from a demanding environment that are difficult to meet, and the threat of failure can evoke intense negative emotions and related physiological responses [13]. A recent review on measures of work stress and related biomarkers has outlined the importance of further research using the ERI model on the basis of its theoretical relevance to the biological stress process and its relevance to recent trends in occupational conditions in the context of economic globalization and rapid technological change [5].

Stress is a biological response of the body to stressors, and two main neuroendocrine stress biological systems are activated in response—the sympathoadrenal system and the hypothalamic pituitary adrenal axis (HPA). There are now a number of studies on the biological stress correlates of work related stress. In a systematic review conducted by Chandola, Heraclides, and Kumari [4], work stress was related to elevated stress responses in terms of sympathoadrenal and HPA axis biomarkers. Eddy, Wertheim, Hale, and Wright [14] also found that the markers of HPA axis activity such as the cortisol awakening response and waking cortisol were associated with ERI, indicating HPA reactivity and responsivity may be altered by work-related stress. Jarczok, Jarczok, Mauss, Koening, Li, Herr, and Thayer [15] found an association between work stress and decreases in neural vagal control of the heart, which indicates a diminished activity of autonomic nervous system response to environmental challenges. A recent systematic review concluded that there were consistent and robust associations between ERI and heart rate variability, altered blood lipids, and risk of metabolic syndrome [5]. Meanwhile, Eddy, Heckenberg, Wertheim, Kent, and Wright [16] found greater workplace stress (ERI) was associated with lower immunity and that there was a significant leukocyte and cytokine response to chronic work stress. Hansen, Larsen Rugulies, Garde, and Knudsen [17] found a robust association between Hba1c, testosterone, and fibrinogen in serum with different dimensions of the psychosocial working environment.

Most of these studies have shown that employees with job-strain, ERI and other measures of adverse psychosocial working conditions have higher biological stress responses, such as increased activation of the HPA axis, metabolic and inflammatory factors. Employees who experience repeated psychosocial stress at work could experience sustained activation of these biological stress responses. The triggering of allostatic load (AL) refers to the cumulative wear and tear that the body experiences when it is not capable of turning-on (beneficial in the short run) or shutting-off (damaging in the long run) repeated cycles of allostasis [18]. It reflects the response pattern of the primary mediators of the neuroendocrine stress response (such as glucocorticoids and catecholamines), which can have protective (such as to adapt and maintain homeostasis) and damaging effects (such as to accelerate disease processes) on the body [19,20]. Allostatic load is typically measured by combining measures of primary mediators and secondary outcomes (sub-clinical disturbances in markers of cardiovascular, metabolic, and immune functioning) [21]. Allostatic load thus provides a measure of chronic “stress”-related physiological processes, showing how the protective and adaptive effects of physiological mediators are involved in the cumulative effects of daily life stressors [22,23]. Allostatic load refers to the cumulative cost to the body of allostasis as individuals adjust their morphology, physiology, and behaviour to unpredictable perturbations [24,25]. It could provide an insight into the way in which individuals respond adequately or inadequately to perturbations of the environment [26] such as chronic work place stressors.

There are a few studies on the association between effort-reward imbalance (ERI) and allostatic load (AL) and other measures of HPA axis activity such as cortisol. For example, Maus, Jarczok, and Fischer [27,28] found that employees exposed to high work stress had higher AL scores, compared to those with low stress levels. They found the same results with two different studies, suggesting a robust association between ERI and AL. Another study by Juster, Sindi, Marin, Perna, Hashemi, Pruessner, and Lupien [29] found that increased AL was associated with increased chronic stress in workplace and burnout symptoms. Bellingrath, Weigl, and Kudielka [30] examined the relationship between work-related chronic work stress and AL. They found that significantly higher levels of AL were found among female school teachers with high levels of ERI.

However, most of these studies used cross-sectional measures of ERI [31,32,33,34,35,36,37,38,39,40] and AL [27,29,41,42,43] which limits any causal inference regarding the direction of relationships between the variables [27,28,31,40]. Furthermore, the proportion of men and women in many studies was highly uneven [27,29,31,34,37,39]. In some of these studies, the cohort was predominantly male with a small proportion of females (or vice-versa). It is particularly important to include female workers in the analysis of work related stressors as they often have worked in the poorest work quality conditions. In some studies, the sample sizes were often quite small [29]. Furthermore, many studies focused on workers belonging to specific occupations (e.g., school teachers, workers, industrial workers), which makes it difficult to generalize from these studies to wider populations of workers.

A review of studies measuring allostatic load in the workforce identified a number of methodological issues [21]. Such review found that there was substantial heterogeneity in the way the concept of allostasis was measured, and many studies did not include primary mediators in the AL index calculation. Given that primary mediators are key features of the AL process, their lack of inclusion could introduce considerable measurement error in AL.

Recent research has shown that older workers at lower grades of employment have more adverse levels of diurnal cortisol profiles in terms of flatter diurnal slopes [44]. As work-related stressors like ERI are more prevalent among lower employment grades [45], it is particularly important to examine how work-related stressors affect chronic stress related biomarkers among older workers, given the context of policies to extend working life policies in many countries. The present study addresses some of the limitations of existing studies because it measures repeated reports of ERI toward the end of the working life course. So, rather than a single measurement of ERI, we measure multiple reports by employees of ERI. We hypothesize that older employees who repeatedly report ERI on more than one occasion (“chronic” or accumulated ERI) are much more likely to experience higher levels of AL than those who are less exposed. Additionally, we examine when in the working life course employees were exposed to ERI. We hypothesize that older employees who were exposed more recently to ERI would have higher levels of AL than those exposed earlier on in their career.

The use of multiple waves of data also allows us to assess temporal factors, such as whether more recent reports of ERI are associated with greater AL [46], as well as allows us to have a better measure of ERI over the working life course. Additionally, we will examine the association between ERI and AL [32], controlling for different factors/covariates, including baseline levels of AL [14,41,47].

The aim of the present study was to examine if repeated reports of work stress over a working career predicts higher levels of allostatic load. We additionally explored different aspects of this association in terms of temporal scheduling of ERI (are more recent experiences of ERI associated with AL compared to experiences further in the past), which dimension of ERI was more salient (increased effort or lower reward), and which AL component was more salient (neuroendocrine, metabolic, inflammatory, cardiovascular systems).

## 2. Materials and Methods

### 2.1. Data

The English Longitudinal Study of Ageing (ELSA) is a panel study of a representative cohort of older men and women living in England aged over 50 years [48]. The biomarker data needed to calculate allostatic load (AL) was first available at wave 2 (2004–2005). At wave 6 (2012–2014), there were additional biomarkers collected which enabled a more detailed measure of AL. The ELSA samples were refreshed at waves 3, 4, and 6 with new entrants to the panel study (aged 50+). All participants provided informed consent separately for the interview and nurse’s visit. Participants gave full informed written consent to participate in the study, and ethical approval was obtained from the National Research Ethics Committee (reference number MREC/01/2/91 and approved on 7 February 2002). Full details of the scoring examinations are reported elsewhere [48].

### 2.2. Variables

#### 2.2.1. Effort-Reward Imbalance

The measurement of effort-reward imbalance (ERI) at work is based on a shortened version of the original ERI questionnaire [49] and combines descriptive and evaluative information on perceived demands (effort) and rewards using indicators that are measured by psychometric scale containing Likert-scale items (where 1 = strongly agree, 2 = agree, 3 = disagree, and 4 = strongly disagree) [36]. Effort was measured by two items (e.g., “My job is physically demanding”, and “I am under constant pressure due to a heavy workload”), while reward was measured by five items (e.g., “I receive the recognition I deserve in my work”, “My salary is adequate”, “My job promotion prospects are poor”, “My job security is poor”, and “I receive adequate support in difficult situations”). ERI was calculated as a ratio of the mean of the effort items to the mean of the reward items. The ERI ratio was then recoded into no imbalance (less than or equal to 0) and imbalance (greater than 1) groups [36,50].

A cumulative ERI score was calculated in the following way: (a) the effort and reward scores were created for each wave, (b) then we created the ERI ratio for each wave. (c) We recoded the ERI ratio values into 0 (for values less than or equal to 0) and 1 (for values greater than 1). (d) Once we had the new recoded binary ERI measure, we summed the ERI scores from each wave (waves 2 to 6) to create the cumulative ERI score. (e) Finally, we recoded the cumulative ERI score into three groups—0 (no occasions of ERI), 1 (one occasion of ERI), and 2 (two or more occasions of ERI).

The cumulative ERI score included anyone who had a measurement of ERI between waves 1–6. By wave 6, only 26% of the sample still had employee status, so the cumulative measure of ERI included those who had exited the labour market by wave 6 (and hence had missing ERI scores) but had previously been employees and had a measurement of ERI. In addition, due to new entrants to ELSA from the refreshment samples and the non-monotonic design of ELSA, only 7.5% of the sample at wave 6 had 5 waves of ERI measurements. 62% of the sample at wave 6 had 2 or more measurements of ERI from wave 2 onwards. In sensitivity analyses, we restricted the analyses of cumulative ERI to those who had at least two measurements of ERI observed between waves 2 and 6.

For the analyses on the specific domains of ERI, the effort and reward scores were grouped into low and high groups at the median cut point [32].

#### 2.2.2. Allostatic Load

Descriptive statistics on each biomarker component of the AL index and the gender specific cut points used to create the overall AL index score are shown in Table 1.

The allostatic load was originally based on data from physiological or physical measurements across neuroendocrine, cardiovascular, metabolic, immune, and anthropometric systems [51]. At wave 6 of ELSA, there were four primary mediators measured that indicate neuroendocrine activity-cortisol and cortisone (from hair samples), insulin growth factor (IGF-1), and pulse rate (which is controlled by the autonomic nervous system). The hair sample collection and analysis are detailed here [52]. As hair analytes were only processed for a subsample of ELSA wave 6 participants with valid hair samples, there were much fewer respondents with cortisol and cortisone data as compared to the other biomarker components of the AL index. Levels of hair cortisone are moderately correlated with hair cortisol (0.3), with both indicating long term HPA axis activity [53].

The immune system was measured using white blood cell count, C-reactive protein (CRP), and fibrinogen level. The metabolic system was measured from the total cholesterol-to-HDL ratio, the glycated haemoglobin level (Hba1c) and triglyceride levels. The cardiovascular system was indicated from systolic and diastolic blood pressure levels and the use of anti-hypertensive medication. The resting blood pressure and pulse rates were taken using Omron machines (after sitting for at least five minutes). The anthropometric system was measured by the waist to height ratio, and the presence of underweight (body mass index < 18.5). Hip measurements are not available in ELSA, so a waist to hip ratio could not be calculated. Moreover, there is some evidence that waist to height ratios are a better predictor of multiple coronary heart disease risk factors [54].

Each of these biomarkers was grouped into high (with the value 1) vs. low (with the value 0) risk groups. Gender specific quartile cut off points were used to define these groups [19,53,55] (see Table 1 for more details), with the exception of underweight (which was defined using BMI < 18.5). Respondents in the highest quartile of the distribution of cortisol, cortisone, pulse rate, white blood cell count, CRP, fibrinogen, total cholesterol-to-HDL ratio, Hba1c, triglyceride, systolic and diastolic blood pressure, and waist to height ratio were defined as being in the risk group, while those in the lowest quartile of IGF-1 were defined as being in the risk group. The allostatic load index was the sum of each of these biomarker risk groups. Respondents had to have at least one observation of a biomarker within a system to contribute to the overall allostatic load index.

At wave 2, there were fewer biomarkers measured, so the allostatic load index was based on measurements of pulse rate, CRP, fibrinogen, total cholesterol-to-HDL ratio, Hba1c, triglycerides, systolic and diastolic blood pressure, hypertensive medication, waist to height ratio, and underweight.

#### 2.2.3. Covariates

Covariates included the following variables: age (categorized as 50–54 years; 55–59 years; 60–64 years; 65–69 years, 70–74 years; and above 75 years), gender (male and female), ethnicity (White British and Non-White British), smoking status (non-smokers vs. current smokers), general health (a subjective measure of self-rate health with five categories: excellent, very good, good, fair, and poor), number of medications used (categorized as 0, 1, 2 and 3 medications or more), depressive symptoms using the Centre for Epidemiologic Studies Depression Scale (with a cut off of four indicating depressive symptoms), physical activity (frequency of moderate to vigorous sports activities with four categories: more than once a week, once a week, one to three times a month, and hardly ever or never), and alcohol use in the last 12 months (categorized as almost every day, five or six days a week, three or four days a week, once or twice a week, once or twice a month, once every couple of months, once or twice a year, and not at all in the last 12 months).

### 2.3. Analytical Sample and Statistical Models

**Figure 1:**
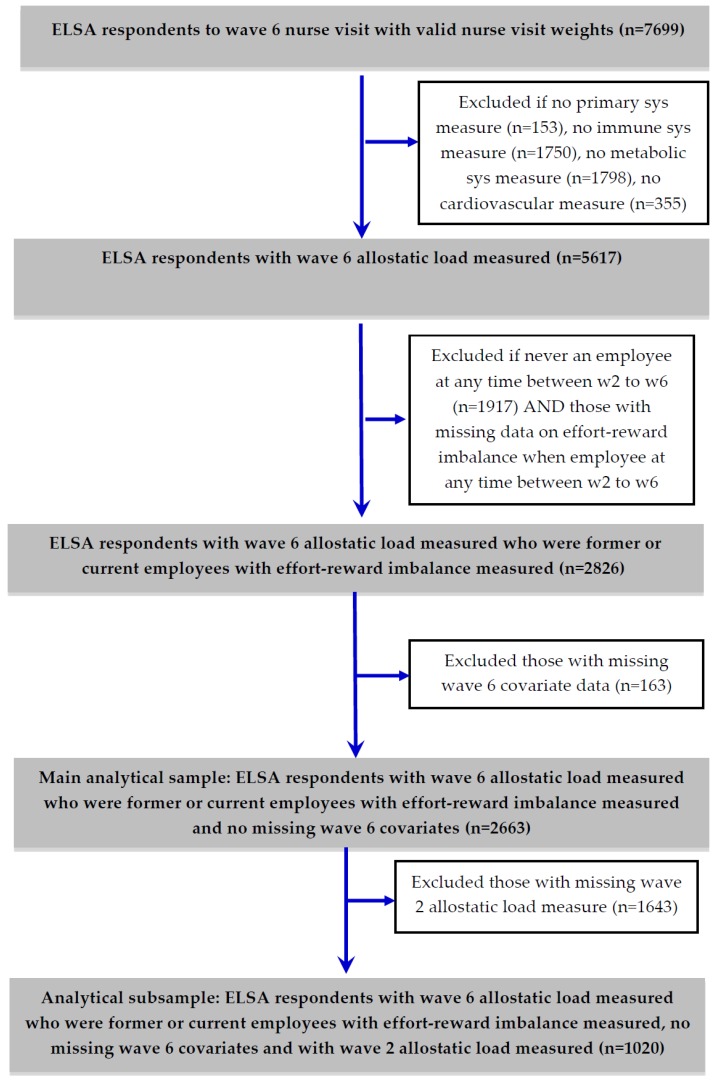
Derivation of main analytical sample and subsample with wave 2 allostatic load measured from English Longitudinal Study of Ageing (ELSA) wave 6 nurse visit sample.

The main analytical sample in this study (see Figure 1) was derived from the ELSA wave 6 nurse visit sample (*n* = 7699), out of which 5617 respondents had their allostatic load index score measured at wave 6. Among this group 2826 respondents were an employee at least once between waves 2 and 6 and had a measurement of ERI when they were an employee. The main analytical sample further reduced to 2663 when missing covariates were deleted from the sample. From this main analytical sample, a subsample of 1020 respondents had their allostatic load measured at wave 2.

As the allostatic load index and the component systems are a count of biomarker risk indicators, the appropriate regression models to model count data include negative binomial regression and Poisson regression models. The allostatic load index-dependent variable was over-dispersed (the variance of allostatic load was greater than the mean), so negative binomial regression models were used to estimate the association between effort-reward imbalance and allostatic load after controlling for covariates. Poisson regression models were used to model the association between the effort-reward imbalance and the neuroendocrine, immune, cardiovascular, and inflammatory systems. A logit model was used for the anthropometric system as the count of the two risk factors (waist-height ratio risk quartile and underweight) reduced to a binary variable in the main analytical sample.

Wave 6 cross-sectional nurse visit survey weights (derived by the ELSA study team) were used to examine the association of cumulative ERI with AL in all the regression models [56]. The wave 6 blood sample survey weights were not used as some of the wave 6 respondents provided a hair sample (for the cortisol and cortisone analytes) but did not provide a blood sample. The longitudinal weights were not appropriate as these have been derived only for core ELSA members from wave 1, and their use would have deleted refreshment sample members from the analysis. All statistics were calculated using the “survey (svy)” command in Stata version 14 (StataCorp., College Station, TX, USA) [57], which takes account of sample selection, non-response bias and the complex survey design for point estimates and variance estimation.

## 3. Results

Table 2 displays the distribution of all the covariates (taken from wave 6) and allostatic load (at wave 6) by cumulative effort-reward imbalance (ERI) for ELSA respondents who had at least ERI measured on at least one occasion between waves 2 and 6. The mean and standard deviations (for continuous covariates) and percentages (for categorical covariates) by cumulative ERI are shown.

The mean level of wave 6 AL index was much higher among ELSA respondents who had reported two or more ERI occasions previously compared to those who did not report any ERI. Women were more likely to report accumulated ERI (two or more occasions of ERI) compared to men. Unsurprisingly, respondents of working age (between 55–64) were more likely to report accumulated ERI. Accumulated ERI tended to occur among those working in semi-routine and routine occupations. ELSA respondents with poorer health (either self-rated health, medication use, or depressive symptoms) were more likely to report accumulated ERI than to not report any ERI.

Table 3 reports the results of the regression models predicting the counts of the overall wave 6 AL index and the risk factors that make up the systems that comprise the wave 6 AL index. Cumulative ERI was significantly associated with the overall AL index (see Supplementary Table S1 for the full model coefficients). Moreover, the coefficients increased in size from no reports to two or more reports of ERI for the component systems of the AL index. However, only the immune system was significantly associated with cumulative ERI. Employees with two or more reports of ERI had a higher estimate of inflammatory factors (0.16, 95% CI: 0.02–0.29) compared with employees who did not report any ERI. In sensitivity analyses, we restricted the analyses of the overall AL index only to those participants who had two or more measurements of ERI from waves 2 to 6. Very similar results were obtained with employees who reported more occasions of ERI having higher estimates of the AL index (see Supplementary Table S2).

Table 4 reports the results of the negative binomial regression models of wave 6 allostatic load predicted by ERI from wave 2 onward. Employees with ERI at the more recent waves 5 (0.09, 95% CI: −0.002–0.17) and 6 (0.13, 95% CI: 0.03–0.22) had higher levels of wave 6 AL compared to those who did not report any imbalance at those waves. In contrast, reports of ERI at earlier waves were not significantly associated with wave 6 AL. None of the separate domains of effort or reward significantly predicted wave 6 AL (analysis not shown), although the effort coefficients were all positive (indicating higher effort was associated with a higher AL index), and the reward coefficients were negative in the most recent waves (indicating lower rewards were associated with a higher AL index).

**Figure 2:**
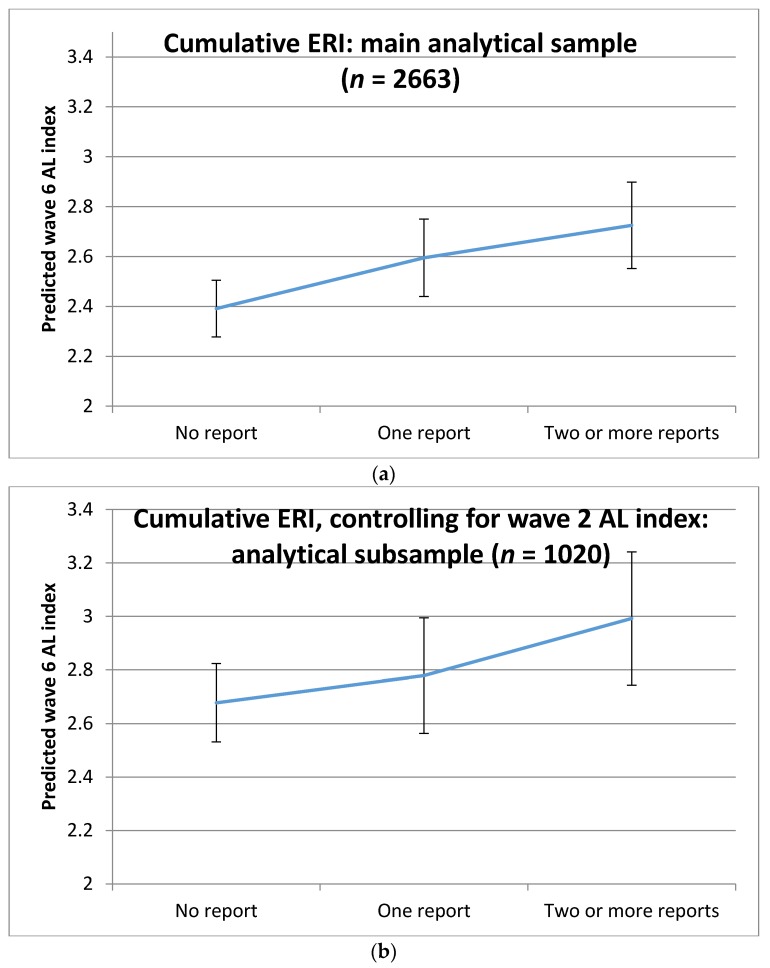
Predicted levels of allostatic load (AL) at wave 6 (and 95% confidence intervals) by cumulative effort-reward imbalance (ERI): (**a**) estimates from main analytical sample (*n* = 2663) displayed in Supplementary Table S1; (**b**) estimates from analytical subsample (*n* = 1020) displayed in Table 5.

Table 5 reports the results of the Poisson regression models of allostatic load by wave 6, controlling for covariates at wave 6 and also controlling for allostatic load measured at wave 2. Employees who reported two or more occasions of ERI had significantly higher estimates counts of the AL index than those who did not report any ERI. In addition, men aged 70–74 and those who took more medications had higher levels of AL. The predicted levels of AL by cumulative reports of ERI from the model not controlling for wave 2 AL (for the main analytical sample as displayed in Supplementary Table S1) is shown in Figure 2a, and controlling for wave 2 Al (for the analytical subsample as displayed in Table 5) is shown in Figure 2b. Both figures clearly show that workers who reported two or more occasions of ERI have around a 0.3 higher estimate of the AL index compared to workers who never reported ERI.

## 4. Discussion

We found some evidence that toward the end of the working career, higher levels of effort-reward imbalance (ERI) predicted higher levels of the allostatic load (AL) index, corresponding to a 0.3 higher estimate of AL index, compared to employees who never reported any imbalance. Moreover, the evidence suggests that employees who had experienced ERI more recently had higher levels of AL compared to those who had experienced ERI earlier in their careers. Among the different AL systems, cumulative ERI was more strongly associated with the immune system compared to the other AL systems. This finding is in accordance with the results reported by Almadi, Cathers, and Chow [55], who found that chronic stress results in increased pro-inflammatory and reduced anti-inflammatory activity. The results also suggested that it was the imbalance between efforts and rewards that was particularly important for AL—the effort and reward dimensions on their own did not significantly predict AL.

The association between work stress and higher levels of AL has been reported in a number of previous studies [27,28,29]. In their recent review on work stress and allostatic load related biomarkers, Siegrist and Li [5] found consistent associations between ERI and reduced heart rate variability, altered blood lipids, and increased markers of metabolic syndrome. There were less consistent findings between ERI and altered catecholamine secretion and elevated fibrinogen. This study has tried to address the one of the main limitations of existing studies highlighted by the review, where in the majority of studies in the review with cross-sectional data, assessment of ERI was restricted to one measurement wave. What is new in this study is the longitudinal finding that higher levels of AL are associated with more reported occasions of ERI, even after taking into account baseline levels of AL. This suggests a dose-response association between repeated reports of ERI and higher levels of AL. A dose-response association adds to the plausibility that exposure to work-related stressors has an effect on biological stress mechanisms, which in turn could lead to incident stress-related disorders, such as coronary heart disease, type 2 diabetes, or depression. Moreover, previous cross-sectional studies that have reported small or inconsistent associations between ERI and related biomarkers may have suffered from inaccurate work stress measurement. With work-related stress being one of the reasons for labour market exit [58], cross-sectional associations between ERI and biological processes linked to health may underestimate the true effect of work-related stressors over a working lifetime on health.

As this is an observational study, we cannot make any causal claims. There may be other confounding factors that we have not taken into account that may explain the association between ERI and AL. For example, sleep problems may be a confounder, although sleep problems could also lie on the causal pathway from work-related stress to AL [59]. ELSA did not measure all the relevant biomarkers needed to construct the AL index, and we lacked some key primary mediators of allostatic load [21], such as cortisol at wave 2. Moreover, the use of quartiles to define risk groups of the AL biomarkers meant that the AL index measure was mainly dependent on the study sample and the availability of specific biomarkers in ELSA. Alternative methods of calculating the AL index could have used clinical risk indicators for some of the biomarkers, but this may result in greater weight to some components of AL than others. For example, there are no existing methods to define very low or high levels of hair cortisol that could be damaging to health. The overcommitment dimension of ERI was also not measured in ELSA. The wave 6 nurse survey weights were used in the analysis, which makes the sample representative of the English older (aged 50+) population, although those survey weights do not take account of the substantial missingness for some of the AL index variables such as cortisol and cortisone.

Despite these limitations, the study has a number of strengths. The use of several waves allows us to explore temporal differences between recent and past reports of ERI. The use of multiple measures of ERI allowed us to estimate ERI over the working life course. The use of longitudinal data from ELSA allowed us to control for baseline levels of AL. The rich ELSA dataset meant that we could control for a number of health and sociodemographic states that could potentially confound the association between ERI and AL. This study showed that repeated reports of ERI over a working life course is associated with higher levels of AL. As ERI has been shown to be related to early labour market exit [58], it is possible that cumulative exposure to work stress is resulting in damaging physiological health changes, which then leads to disability related labour market exit. Reducing work-related stress could improve the employment patterns and success of older workers.

## 5. Conclusions

Although there are a number of cross-sectional studies on the association between ERI and stress-related biomarkers, there is little or no evidence on whether the repeated exposure to work stress over a working career predicts higher levels of AL. The study finds some evidence that older adults aged 50+ living in England who repeatedly reported ERI had higher levels of the AL index than those who did not report any ERI. This association was robust to controlling for a range of potential health and socio-demographic confounders, as well as baseline levels of AL. Furthermore, more recent reports of ERI were associated with higher levels of AL, suggesting plausible links between the timing of the stressor and the stress response. The findings of a dose-response association between ERI and AL, as well as the timing of the stressor and stress response, suggest that exposure to work-related stressors may have adverse consequences for physiological health through increasing adverse levels of stress related biomarkers.

## Figures and Tables

**Table 1:** Distribution of the allostatic load index component biomarkers at waves 2 and 6.

				Cut off for AL Index	
Wave 6 Allostatic Load Biomarkers	Mean/%	SD	*n*	Women	Men
**Neuroendocrine system**					
Cortisol (pg/mL)	46.9	201.9	947	>21.9	>18.5
Cortisone (pg/mg)	8.5	9.0	956	>7.6	>11.5
Insulin growth factor 1 (nmol/L)	17.1	4.9	2640	<13	<15
Pulse rate (beats per minute)	54.7	12.2	2638	>67.5	>66.5
**Immune system**					
White blood cell (×10^9^ cells/L)	6.3	1.8	2614	>7.42	>7.63
C-reactive protein (mg/L)	2.0	1.9	2539	>3	>2.6
Fibrinogen (g/L)	2.9	0.5	2561	>3.3	>3.2
**Metabolic system**					
Total cholesterol to HDL ratio	3.6	1.1	2648	>3.89	>4.46
Triglycerides (mmol/L)	1.5	0.9	2650	>1.7	>1.9
Hba1c (%)	40.0%	7.1	2613	>42	>43
**Cardiovascular system**					
Systolic blood pressure (mmHg)	132.6	17.2	2638	>145.5	>147
Diastolic blood pressure (mmHg)	76.9	10.2	2638	>81.5	>82.5
Anti-hypertensive medication	21.0%	-	2663		
**Anthropometric system**					
Waist to height ratio	0.6	0.1	2641	>0.6325	>0.6327
Underweight (%)	0.7%	-	2628	<18.5	<18.5
**Wave 6 allostatic load index**	2.6	2.0	2663		
**Wave 2 allostatic load biomarkers**					
**Neuroendocrine system**					
Pulse rate (beats per minute)	54.1	11.5	1020	>69	>68
**Immune system**					
C-reactive protein (mg/L)	2.1	2.1	968	>3.7	>3.2
Fibrinogen (g/L)	3.0	0.6	999	>3.7	>3.5
**Metabolic system**					
Total cholesterol to HDL ratio	4.0	1.0	1013	>4.44	>4.85
Triglycerides (mmol/L)	1.8	1.2	1014	>2.1	>2.3
Hba1c (mmol/mol)	36.1	6.2	1007	>38.8	>39.9
**Cardiovascular system**					
Systolic blood pressure (mmHg)	132.2	17.3	1020	>149	>148
Diastolic blood pressure (mmHg)	77.7	10.7	1020	>82.5	>84
Anti-hypertensive medication	8.2%	-	1020		
**Anthropometric system**					
Waist to height ratio	0.6	0.1	1009	>0.6214	>0.6289
Underweight (%)	0.5%	-	1003	<18.5	<18.5
**Wave 2 allostatic load index**	1.7	1.6	1020		

**Table 2:** Distribution of wave 6 allostatic load (AL) index and covariates by cumulative effort-reward imbalance among ELSA participants.

Wave 6 Variables	Cumulative Effort-Reward Imbalance		
	No Report	One Report	Two or More Reports
	Mean (SD)/%	Mean (SD)/%	Mean (SD)/%
*n*	1403	717	543
**AL index mean (SD)**	2.45 (1.90)	2.63 (2.07)	2.91 (1.97)
**Gender**			
Men	53%	52%	47%
Women	47%	48%	53%
**Age groups**			
50–54	10%	17%	1%
55–59	21%	26%	36%
60–64	28%	29%	40%
65–69	26%	20%	19%
70–74	11%	6%	3%
75+	4%	2%	1%
**Ethnicity**			
White British	97%	98%	97%
Non-white	3%	2%	3%
**Social class**			
Professional	44%	40%	32%
Intermediate	15%	11%	11%
Small employers	14%	14%	10%
Lower & technical	5%	10%	13%
Semi-routine & routine	21%	25%	34%
**Employment status**			
Employed	52%	60%	71%
Retired	44%	35%	28%
Disabled/looking after family	4%	5%	2%
**Current smoker**			
No	91%	88%	88%
Yes	9%	12%	12%
**Self-reported health**			
Excellent/good	90%	85%	78%
Fair/poor	10%	15%	22%
**Number of medications**			
0 meds.	39%	35%	32%
1–2 meds.	30%	32%	33%
3–5 meds.	22%	22%	25%
≥6 meds.	9%	10%	10%
**Depressive symptoms**			
CESD score < 4	95%	91%	87%
CESD score ≥ 4	5%	9%	13%
**Vigorous physical activity**			
<once a week	30%	27%	28%
Once a week	12%	10%	13%
1–3 times a month	12%	12%	11%
Never	47%	51%	48%
**Moderate physical activity**			
<once a week	73%	73%	74%
Once a week	16%	14%	13%
1–3 times a month	5%	6%	6%
Never	5%	8%	7%
**Alcohol consumption**			
Almost every day	16%	14%	14%
5–6 days a week	8%	6%	6%
3–4 days a week	20%	18%	13%
1–2 a week	24%	29%	28%
1–2 a month	12%	13%	14%
Once in 2 months	7%	6%	6%
1–2 times a year	6%	8%	9%
Never	7%	5%	9%

**Table 3:** Survey weighted regression model coefficient estimates (and 95% CI) of wave 6 allostatic load systems regressed on cumulative effort-reward imbalance.

Wave 6 Allostatic Load (AL) System	Cumulative Effort-Reward Imbalance (Ref.: No Report)	
	One Report	Two or More Reports
	**Coeff. (95% CI)**	**Coeff. (95% CI)**
Sympathoadrenal ^1,2^	0.09 (−0.04, 0.22)	0.11 (−0.02, 0.24)
*p*-value	0.187	0.105
Immune ^1,2^	0.11 (−0.02, 0.24)	0.16 (0.02, 0.29)
*p*-value	0.107	0.025
Metabolic ^1,2^	0.08 (−0.05, 0.21)	0.10 (−0.04, 0.23)
*p*-value	0.206	0.152
Cardiovascular ^1,2^	0.01 (−0.11, 0.13)	0.11 (−0.01, 0.23)
*p*-value	0.859	0.081
Anthropometric ^1,3^	−0.05 (−0.30, 0.21)	0.06 (−0.21, 0.33)
*p*-value	0.727	0.670
Overall AL index ^1,4^	0.08 (0.003, 0.16)	0.13 (0.05, 0.21)
*p*-value	0.042	0.001

^1^ Models control for all the covariates listed in Table 2, ^2^ Poisson model, ^3^ logit model, and ^4^ negative binomial regression models.

**Table 4:** Survey weighted negative binomial regression coefficients (and 95% CI) of wave 6 allostatic load index regressed on effort-reward imbalance, effort, and reward at ELSA waves 2–6 ^1^.

ERI Measured at	Coeff. (95% CI)	*p*-Value
Wave 2	0.03 (−0.06, 0.12)	0.476
Wave 3	−0.03 (−0.12, 0.06)	0.487
Wave 4	0.04 (−0.05, 0.12)	0.368
Wave 5	0.09 (−0.002, 0.17)	0.055
Wave 6	0.13 (0.03, 0.22)	0.008

^1^ Models control for all the covariates listed in Table 2.

**Table 5:** Survey weighted Poisson regression coefficients (and 95% CI) of wave 6 allostatic load index regressed on cumulative effort-reward imbalance, wave 6 covariates, and wave 2 allostatic load index: ELSA analytical subsample (*n* = 1020).

	Coeff. (95% CI)	*p*-Value		Coeff. (95% CI)	*p*-Value
**Cumulative ERI (Ref.: No report of ERI)**			**Vigorous physical activity (Ref.: <once a week)**		
One report of ERI	0.04 (−0.06, 0.13)	0.434	Once a week	0.06 (−0.08, 0.19)	0.423
Two or more reports of ERI	0.11 (0.01, 0.22)	0.037	1–3 times a month	0.06 (−0.09, 0.22)	0.417
AL index at W2	0.18 (0.16, 0.21)	<0.001	Never	0.06 (−0.05, 0.17)	0.289
**Socio-economic classification (Ref.: Professional)**			**Moderate physical activity (Ref.: <once a week)**		
Intermediate	0.04 (−0.09, 0.17)	0.577	Once a week	0.08 (−0.03, 0.19)	0.141
Small employers	0.09 (−0.04, 0.22)	0.160	1–3 times a month	0.15 (−0.01, 0.30)	0.067
Lower & technical	0.05 (−0.10, 0.20)	0.529	Never	0.07 (−0.08, 0.23)	0.340
Semi-routine & routine	0.08 (−0.02, 0.19)	0.132	**Alcohol consumption (Ref.: Almost every day)**		
**Employment status (Ref.: Employed)**			5–6 days a week	−0.11 (−0.29, 0.08)	0.271
Retired	−0.02 (−0.11, 0.08)	0.721	3–4 days a week	−0.07 (−0.21, 0.08)	0.371
Sick-Disable/family carer	0.04 (−0.13, 0.21)	0.659	1–2 a week	−0.09 (−0.22, 0.04)	0.170
**Gender (Ref.: Women)**			1–2 a month	0.03 (−0.12, 0.18)	0.682
Men	−0.13 (−0.26, −0.01)	0.040	Once in 2 months	−0.03 (−0.22, 0.16)	0.742
Age (Model 1: Ref.: 50–54) (Model 2: Ref.: Men * 60–64)			1–2 times a year	0.11 (−0.05, 0.27)	0.176
55–59			Never	0.03 (−0.12, 0.18)	0.690
60–64	0.06 (−0.06, 0.18)	0.316	**Depressive symptoms (Ref.: CESD score < 4)**		
65–69	−0.12 (−0.30, 0.06)	0.204	CESD score ≥ 4	−0.05 (−0.21, 0.12)	0.590
70–74	0.01 (−0.27, 0.29)	0.957	Intercept	0.42 (0.23, 0.62)	<0.001
75+					
Gender * Age (Model 1: Ref.: 50–54) (Model 2: Ref.: Men * 60–64)					
Men aged 55–59					
Men aged 60–64					
Men aged 65–69	0.04 (−0.14, 0.22)	0.636			
Men aged 70–74	0.39 (0.15, 0.63)	0.001			
Men aged 75+	0.30 (−0.05, 0.66)	0.091			
**Ethnicity (Ref.: White British)**					
Non-White ethnic group	0.10 (−0.17, 0.37)	0.487			
**Current smoker (Ref.: No)**					
Yes	0.07 (−0.06, 0.21)	0.279			
**Self-reported Health (Ref.: Excellent/good)**					
Fair/poor	0.07 (−0.04, 0.18)	0.243			
**Number of medications (Ref.: 0 meds.)**					
1–2 meds.	0.11 (−0.01, 0.22)	0.079			
3–5 meds.	0.16 (0.04, 0.29)	0.012			
≥6 meds.	0.19 (0.04, 0.34)	0.012			

* Interaction term of gender and age-group.

## Supplementary Materials

The following are available online at http://www.mdpi.com/1660-4601/15/2/191/s1, Table S1. Survey-weighted negative binomial regression coefficients (and 95% CI) of wave 6 allostatic load index regressed on cumulative effort-reward imbalance and wave 6 covariates: English Longitudinal Study of Ageing (ELSA) main analytical sample (*n* = 2663), Table S2. Survey-weighted negative binomial regression coefficients (and 95% CI) of wave 6 allostatic load index regressed on cumulative effort-reward imbalance (at least two occasions of ERI observed) and wave 6 covariates: ELSA sensitivity analyses (*n* = 1871).
