# Supplementary material for: Allostatic Load and Effort-Reward Imbalance: Associations over the Working-Career
Source: Int J Environ Res Public Health. 2018 Jan 24;15(2):191. doi: 10.3390/ijerph15020191 (PMC5857048; doi:10.3390/ijerph15020191)
Supplement: Supplementary file 1 [file ijerph-15-00191-s001.zip › ijerph-253086 supplementary 2 for proofreading.docx]

**Table S2.** Survey-weighted negative binomial regression coefficients (and 95% CI) of wave 6 allostatic load index regressed on cumulative effort-reward imbalance (at least two occasions of ERI observed) and wave 6 covariates: ELSA sensitivity analyses (*n*= 1,871).

| **Coeff. (95% CI)** | | ***p*-value** | **Coeff. (95% CI)** | | ***p*-value** |
| --- | --- | --- | --- | --- | --- |
| **Cumulative ERI (Ref: No report of ERI)** | |  | **Vigorous physical activity (Ref: < once a week)** | | |
| One report of ERI |  |  | Once a week | 0.09 (−0.04, 0.21) | 0.173 |
| Two or more reports of ERI | 0.02 (−0.06, 0.11) | 0.598 | 1–3 times a month | 0.20 (0.08, 0.32) | 0.001 |
| AL index at W2 | 0.09 (0.01, 0.17) | 0.031 | Never | 0.19 (0.10, 0.27) | < 0.001 |
| **Socio-Economic Classification (Ref: Professional)** | |  | **Moderate physical activity (Ref: < once a week)** | | |
| Intermediate | 0.01 (−0.09, 0.12) | 0.809 | Once a week | 0.04 (−0.05, 0.13) | 0.357 |
| Small employers | 0.10 (−0.02, 0.21) | 0.103 | 1-3 times a month | 0.13 (−0.02, 0.29) | 0.099 |
| Lower & technical | −0.12 (−0.26, 0.02) | 0.082 | Never | 0.08 (−0.05, 0.21) | 0.243 |
| Semi-routine & routine | 0.03 (−0.06, 0.11) | 0.563 | **Alcohol consumption (Ref: Almost every day)** | | |
| **Employment Status (Ref: Employed)** | |  | 5–6 days a week | −0.12 (−0.28, 0.03) | 0.122 |
| Retired | −0.03 (−0.11, 0.05) | 0.479 | 3–4 days a week | −0.04 (−0.15, 0.08) | 0.537 |
| Sick-Disable/Family carer | 0.01 (−0.17, 0.19) | 0.928 | 1–2 a week | −0.06 (−0.17, 0.05) | 0.320 |
| **Gender (Ref: Women)** |  |  | 1–2 a month | 0.06 (−0.07, 0.18) | 0.369 |
| Men | 0.14 (−0.35, 0.63) | 0.570 | Once in 2 months | 0.16 (0.01, 0.30) | 0.031 |
| **Age (Model 1: Ref: 50**–**54) (Model 2: Ref: Men*60**–**64)** | | | 1–2 times a year | 0.13 (−0.03, 0.28) | 0.101 |
| 55-59 | 0.04 (−0.23, 0.31) | 0.761 | Never | 0.13 (−0.01, 0.26) | 0.067 |
| 60-64 | 0.11 (−0.16, 0.38) | 0.434 | **Intercept** | 0.34 (0.06, 0.63) | 0.017 |
| 65-69 | 0.20 (−0.08, 0.47) | 0.156 | **Alpha** | 0.07 (0.05, 0.12) |  |
| 70-74 | 0.19 (−0.12, 0.5) | 0.230 |  |  |  |
| 75+ | 0.09 (−0.4, 0.59) | 0.706 |  |  |  |
| **Gender*Age (Model 1: Ref: 50**–**54) (Model 2: Ref: Men*60**–**64)** | | | |  |  |
| Men aged 55–59 | −0.03 (−0.54, 0.47) | 0.894 |  |  |  |
| Men aged 60–64 | −0.10 (−0.60, 0.40) | 0.689 |  |  |  |
| Men aged 65–69 | −0.17 (−0.67, 0.33) | 0.510 |  |  |  |
| Men aged 70–74 | −0.09 (−0.64, 0.46) | 0.745 |  |  |  |
| Men aged 75+ | 0.14 −0.54, 0.81) | 0.691 |  |  |  |
| **Ethnicity (Ref: White British)** |  |  |  |  |  |
| Non-White ethnic group | −0.04 (−0.19, 0.11) | 0.607 |  |  |  |
| **Current smoker (Ref: No)** |  |  |  |  |  |
| Yes | 0.20 (0.10, 0.3) | < 0.001 |  |  |  |
| **Self-reported Health (Ref: Excellent/Good)** | |  |  |  |  |
| Fair/Poor | 0.18 (0.09, 0.27) | < 0.001 |  |  |  |
| **Number of Medications (Ref: 0 meds.)** | |  |  |  |  |
| 1-2 meds. | 0.24 (0.15, 0.33) | < 0.001 |  |  |  |
| 3-5 meds. | 0.41 (0.32, 0.51) | < 0.001 |  |  |  |
| ≥ 6 meds. | 0.55 (0.44, 0.67) | < 0.001 |  |  |  |
| **Depressive symptoms (Ref: CESD score < 4)** | |  |  |  |  |
| CESD score ≥ 4 | 0.03 (−0.09, 0.14) | 0.669 |  |  |  |
