# Supplementary material for: Allostatic Load and Effort-Reward Imbalance: Associations over the Working-Career
Source: Int J Environ Res Public Health. 2018 Jan 24;15(2):191. doi: 10.3390/ijerph15020191 (PMC5857048; doi:10.3390/ijerph15020191)
Supplement: Supplementary file 1 [file ijerph-15-00191-s001.zip › ijerph-253086 supplementary for proofreading.docx]

**Table S1.** Survey-weighted negative binomial regression coefficients (and 95% CI) of wave 6 allostatic load index regressed on cumulative effort-reward imbalance and wave 6 covariates: English Longitudinal Study of Ageing (ELSA) main analytical sample (n=2,663).

| **Coeff. (95% CI)** | | ***p*-value** | **Coeff. (95% CI)** | | ***p*-value** |
| --- | --- | --- | --- | --- | --- |
| **Cumulative ERI (Ref: No report of ERI)** | |  | **Vigorous physical activity (Ref: < once a week)** | | |
| One report of ERI | 0.08 (0.003, 0.16) | 0.042 | Once a week | 0.13 (0.01, 0.26) | 0.040 |
| Two or more reports of ERI | 0.13 (0.05, 0.21) | 0.001 | 1–3 times a month | 0.21 (0.08, 0.33) | 0.001 |
| **Socio-Economic Classification (Ref: Professional)** | |  | Never | 0.20 (0.11, 0.29) | < 0.001 |
| Intermediate | 0.02 (−0.09, 0.13) | 0.752 | **Moderate physical activity (Ref: <once a week)** | | |
| Small employers | −0.01 (−0.11, 0.10) | 0.888 | Once a week | 0.04 (−0.05, 0.13) | 0.339 |
| Lower & technical | −0.08 (−0.20, 0.05) | 0.219 | 1–3 times a month | 0.03 (−0.12, 0.17) | 0.728 |
| Semi-routine & routine | 0.02 (−0.06, 0.10) | 0.628 | Never | 0.13 (0.01, 0.25) | 0.032 |
| **Employment Status (Ref: Employed)** | |  | **Alcohol consumption (Ref: Almost every day)** | | |
| Retired | −0.05 (−0.12, 0.02) | 0.169 | 5–6 days a week | −0.04 (−0.19, 0.10) | 0.564 |
| Sick-Disable/Family carer | 0.01 (-0.14, 0.16) | 0.891 | 3–4 days a week | 0.01 (−0.10, 0.12) | 0.842 |
| **Gender (Ref: Women)** |  |  | 1–2 a week | −0.02 (−0.12, 0.08) | 0.679 |
| Men | 0.30 (0.1, 0.50) | 0.003 | 1–2 a month | 0.05 (−0.07, 0.17) | 0.424 |
| **Age (Model 1: Ref: 50–54) (Model 2: Ref: Men*60–64)** | | | Once in 2 months | 0.15 (0.01, 0.30) | 0.042 |
| 55–59 | 0.12 (−0.06, 0.30) | 0.182 | 1–2 times a year | 0.14 (0.01, 0.28) | 0.042 |
| 60–64 | 0.28 (0.11, 0.45) | 0.001 | Never | 0.10 (-0.03, 0.23) | 0.137 |
| 65–69 | 0.35 (0.17, 0.53) | 0.000 | **Intercept** | 0.12 (−0.06, 0.29) | 0.187 |
| 70–74 | 0.35 (0.14, 0.55) | 0.001 | **Alpha** | 0.10 (0.07, 0.14) |  |
| 75+ | 0.26 (−0.06, 0.58) | 0.112 |  |  |  |
| **Gender*Age (Model 1: Ref: 50–54) (Model 2: Ref: Men*60–64)** | | | |  |  |
| Men aged 55–59 | −0.13 (−0.36, 0.11) | 0.29 |  |  |  |
| Men aged 60–64 | −0.27 (−0.49, −0.05) | 0.018 |  |  |  |
| Men aged 65–69 | -0.33 (-0.56, -0.1) | 0.005 |  |  |  |
| Men aged 70–74 | −0.19 (−0.46, 0.08) | 0.161 |  |  |  |
| Men aged 75+ | −0.04 (−0.41, 0.33) | 0.835 |  |  |  |
| **Ethnicity (Ref: White British)** |  |  |  |  |  |
| Non-White ethnic group | 0.07 (−0.13, 0.26) | 0.494 |  |  |  |
| **Current smoker (Ref: No)** |  |  |  |  |  |
| Yes | 0.21 (0.11, 0.31) | < 0.001 |  |  |  |
| **Self-reported Health (Ref: Excellent/Good)** | |  |  |  |  |
| Fair/Poor | 0.15 (0.07, 0.24) | 0.001 |  |  |  |
| **Number of Medications (Ref: 0 meds.)** | |  |  |  |  |
| 1–2 meds. | 0.25 (0.16, 0.33) | < 0.001 |  |  |  |
| 3–5 meds. | 0.50 (0.40, 0.59) | < 0.001 |  |  |  |
| ≥ 6 meds. | 0.58 (0.47, 0.68) | < 0.001 |  |  |  |
| **Depressive symptoms (Ref: CESD score < 4)** | |  |  |  |  |
| CESD score ≥ 4 | 0.03 (−0.09, 0.15) | 0.645 |  |  |  |
